# Identification of QTLs and Putative Candidate Genes for Plant Architecture of Lotus Revealed by Regional Association Mapping

**DOI:** 10.3390/plants12061221

**Published:** 2023-03-08

**Authors:** Mei Zhao, Jibin Zhang, Chuxuan Yang, Zhenhua Cui, Longqing Chen

**Affiliations:** 1 College of Landscape and Forestry, Qingdao Agricultural University, Qingdao 266109, China; 2 College of Horticulture, Qingdao Agricultural University, Qingdao 266109, China; 3 Southwest Landscape Architecture Engineering Research Center (National Forestry and Grassland Administration), Southwest Forestry University, Kunming 650224, China

**Keywords:** lotus, plant architecture, QTLs, SSR markers, InDel markers

## Abstract

The lotus (*Nelumbo* Adans.) is one of the most economically relevant ornamental aquatic plants. Plant architecture (PA) is an important trait for lotus classification, cultivation, breeding, and applications. However, the underlying genetic and molecular basis controlling PA remains poorly understood. In this study, an association study for PA-related traits was performed with 93 genome-wide microsatellite markers (simple sequence repeat, SSR) and 51 insertion–deletion (InDel) markers derived from the candidate regions using a panel of 293 lotus accessions. Phenotypic data analysis of the five PA-related traits revealed a wide normal distribution and high heritability from 2013 to 2016, which indicated that lotus PA-related traits are highly polygenic traits. The population structure (Q-matrix) and the relative kinships (K-matrix) of the association panels were analyzed using 93 SSR markers. The mixed linear model (MLM) taking Q-matrix and K-matrix into account was used to estimate the association between markers and the traits. A total of 26 markers and 65 marker–trait associations were identified by considering associations with *p* < 0.001 and Q < 0.05. Based on the significant markers, two QTLs on Chromosome 1 were identified, and two candidate genes were preliminarily determined. The results of our study provided useful information for the lotus breeding aiming at different PA phenotypes using a molecular-assisted selection (MAS) method and also laid the foundation for the illustration of the molecular mechanism underlying the major QTL and key markers associated with lotus PA.

## 1. Introduction

Lotus (*Nelumbo* Adans.) is a perennial aquatic plant and positioned as a basal eudicot in evolution. As the only genus of Nelumbonaceae family, *Nelumbo* comprises two species, *N. nucifera* and *N. lutea* [1], which can be interbred regardless of the geographical isolation by the Pacific Ocean. In Asia, lotus plays a vital role in cultural and religious activities and is also an economically important crop used for food and medicinal purposes [2]. In addition, lotus is widely used as an important aquatic ornamental plant for its attractive flower features and elegant plant architecture. Moreover, recent studies have shown that lotus also plays an important role in water pollution control, water heavy metal reduction, and water eutrophication alleviation [3,4,5]. Hence, the versatility of lotus determines its importance and popularity [1].

Plant architecture (PA) is the three-dimensional organization of the above-ground plant parts and encompasses, e.g., plant height, branching pattern, the arrangement of leaves and fruit branches, and so on. Similar to most plants, plant height is the decisive factor of PA for lotus [2]. Plant height of lotus is mainly measured as the height of the petiole and peduncle due to its unique morphological structure. In addition, the leaf size and flower size are also important factors in determining PA of lotus [2]. A lotus with small architecture (SA) in a pot shows delicate and exquisite gestures and has become more and more popular in home gardening in recent years [1]. However, as an emergent plant, the normal growth of lotus is influenced seriously by water depth. Lotuses with small and medium architecture are only adapted to 20–60 cm of water depth, and even the lotus with large architecture (LA) cannot grow well in the water with a depth of 1.2 m or greater [1]. However, it is very common that the water depth of natural waters is beyond the upper limit for lotus survival, which would be aggravated during the wet period and floods period. This enhances the demand for lotus varieties with LA, which can cope with the severe challenge of deep water. Hence, breeding lotus varieties with a special size of PA is very important for the decoration in courtyards and public areas and its living in natural waters.

A conventional breeding program is time-consuming and easily affected by the environmental factors [6]. In contrast, using molecular biotechnologies can shorten the breeding cycle and efficiently obtain the desired feathers of the ornamental plants. However, lacking an effective genetic transformation system impedes the application of reverse genetic approaches in lotus breeding, making marker-assisted selection (MAS) a preferential and feasible means to accelerate lotus breeding. Markers linked to the desired traits are the prerequisite to efficiently select the target traits using MAS and to further identify the key genes that specifically control PA of lotus. However, as far as we know, there have been no reports on the quantitative trait loci (QTLs) mapping for PA in lotus, and the markers associated with PA are very rare, which restrains the application of MAS in lotus breeding.

PA in many plants are complex quantitative traits that are controlled and regulated by many minor effect QTLs [7]. To identify the QTLs or genes controlling complex quantitative traits in plants, the linkage mapping and association mapping are the two most commonly used methods [8]. Association mapping takes advantage of the numerous ancestral recombination events accumulated within the natural populations to dissect the associations between the genotype and the phenotype, while linkage analysis uses the limited chromosome exchange within the constructed biparental population to localize the target QTLs or genes [9]. Therefore, association analysis takes less time and can achieve a higher resolution of QTL mapping [10]. However, the presence of population structure and kinship relatedness among accessions used in the research can introduce false-positive marker–trait associations [9,11]. Hence, the data assessing population structure and kinship relatedness were brought into association statistical models as a covariate to decrease the spurious marker–trait association to a certain extent [11,12].

According to the coverage of molecular markers, the strategies of association analysis are mainly divided into two types, genome-wide association analysis (GWAS) and candidate gene/region association analysis. GWAS using molecular markers widely distributing in the whole genome can lay the foundation for subsequent fine mapping and key gene discovery. To date, GWAS has been widely applied in maize [13,14], cotton [15,16], sorghum [17], and soybean [18] for detection of QTLs associated with PA traits. In lotus, association analysis based on 98 genomic-SSR markers and 129 lotus accessions identified that 11 SSR markers were associated with PA-related traits, among which 7 SSR markers were associated with petiole height, 5 SSRs were associated with peduncle height, 6 SSRs were associated with leaf length, and 7 SSRs were associated with leaf width [19]. However, this is still far from mapping the specific QTLs or identifying the key genes. In contrast, candidate gene/region association analysis utilizes molecular markers in the preliminary identified regions or putative genes to narrow down the candidate regions or identify the target genes. For example, five candidate genes regulating starch content within primary mapped QTLs in maize were identified based on the regional association study [20]. In soybean, a regional association study identified a sugar transporter gene from the 4 Mb candidate QTL associated with protein and oil on chromosome 15 [21]. However, the reports utilizing regional association analysis to fine map the PA traits have not been documented.

The access of the whole genome sequence of two lotus germplasms (‘Chinese Tai-zi’ and ‘China Antique’) provides rich genetic information for new molecular markers mining. The total length of the ‘Chinese Tai-zi’ and ‘China Antique’ pseudo-chromosomes is approximately 782.76 Mb and 797.68 Mb, respectively. Sequences of both germplasms are assembled into eight chromosomes and were functionally annotated [22]. SSRs are considered to be the ideal markers for MAS breeding, as they are codominant and hypervariable to distinguish the diversity of the genotypes [23]. Additionally, enormous amounts of single-nucleotide polymorphisms (SNPs) and InDel loci among lotus genotypes have been identified in recent years [24,25,26]. Compared to the high cost of re-sequencing, SSRs can provide an attractive alternative. In addition, SSRs were more effective than SNPs for analyzing maize population structures due to their more variable polymorphism [27]. In lotus, Yang et al. [28] designed 500 SSR primers from the genome sequence, from which 386 pairs produced scorable alleles. These findings provide an important basis for the follow-up mapping work.

In our previous work, we identified a total of 11.02 Mb genomic regions that were highly differentiated between the LA and SA lotus based on whole-genome resequencing data [29]. Within these regions, we identified 17,154 SNPs and 1554 InDels which showed distinct allelic distribution between the two lotus groups. However, more efforts are still needed to explore new markers associated with PA traits and to fine map the loci associated with PA traits, which would be valuable for lotus breeding and research. Based on that, we developed InDel markers in the candidate regions and performed regional association analysis in a set of 293 genotypes in this study. Therefore, the objective of the present study was to (1) assess the genetic diversity of the candidate regions related to PA traits in lotus based on the InDel markers, (2) narrow down the candidate regions and identify key loci for PA related traits, and (3) identify the genes potentially controlling the lotus PA. These results provided valuable information for understanding the genetic basis of PA-related traits and will help in lotus breeding for desired PA traits using MAS.

## 2. Materials and Methods

### 2.1. Plant Materials and Phenotypic Characterization

A collection of 293 lotus accessions, including 237 *N. nucifera* and 56 interspecific hybrids (*N. nucifera* × *N. lutea*) (Table S1), from China lotus research center (Wuhan, Hubei Province, China) were used for association mapping. These accessions are derived from geographically different origins and exhibit a high degree of phenotypic diversity in PA, flower-, and leaf-related traits (Table S1). Each accession was planted in a separate cement pool (2.1 m × 1.2 m × 0.3 m) for the convenience of phenotyping in the experiment. The flower and its accompanying leaf were selected for the measurement of petiole height (PTH) and peduncle height (PDH). The PTH was measured from the soil surface to the joint of petiole and leaf, and the PDH was measured from the soil surface to the joint of peduncle and flower. Leaf length (LL) was measured as the maximum length on the leaf horizontal axis, and leaf width (LW) was measured as the maximum length on the leaf vertical axis. Flower diameter (FD) was measured as the maximum width of the flower. All the five PA-related parameters were examined at the stage of full bloom, and at least 10 randomly picked plants were measured for each parameter.

The association panel was field-evaluated annually according to the five PA-related parameters during June to August from 2013 to 2016. Finally, the mean value of each trait for each accession was used as the phenotyping value of each year. Descriptive statistics, including frequency distribution, mean values, coefficient of variability (CV), Pearson’s correlation coefficients, and inter-annual difference, were performed with SPSS version 19.0. Normality tests of the phenotypic data of the five traits in each year were performed under the D’Agostino–Pearson omnibus normality test (α = 0.05) using GraphPad Prism 8.

### 2.2. Molecular Marker Characterization, Genotyping, and Genetic Diversity Analysis

Total DNA was extracted from fresh young leaves of each lotus genotype using CTAB according to the method previously described [30]. Two subsets of markers were used for polymorphism screening among the association panel individuals. The first group included 300 genomic-SSR markers retrieved from an early report [28]. Ten randomly picked lotus genotypes were used to evaluate the polymorphism of the 300 SSR markers. The SSR amplification reaction was conducted as described by Cao [31]. Polymerase chain reaction (PCR) amplification was carried out in a total volume of 15 µL containing 1 µL template DNA (50 ng/µL), 1.2 µL of each primer (10 µM), 7 µL 2 × Es Taq MasterMix for PAGE (CWBIO, Beijing, China), and 4.6 µL ddH_2_O. PCR amplifications were performed on a 9902 Thermal Cycler (ABI, Waltham, MA, USA) under the following cycle profile: (1) denaturation at 94 °C for 5 min; (2) 30 cycles of denaturation at 94 °C for 30 s, annealing at corresponding Tm of each primer at 72 °C for 30 s, and extension at 72 °C for 30 s; and (3) final extension at 72 °C for 10 min. All the PCR products were separated by electrophoresis using 6% polyacrylamide gels, and the fragments were visualized by silver staining. The allele size was estimated using a DNA ladder with 20 bp intervals (Dongsheng Biotech, Guangzhou, China). The SSR markers that generated clear and polymorphic bands were selected for subsequent experiment. The second group consisted of 51 InDel markers developed from the candidate regions, which were putatively associated with PA of lotus. For the 51 InDel makers, 31 of them were used in the previous study [29], and the other 20 were newly developed using the same method as before [29]. Then, all of the SSR and InDel markers that screened out above were used for genotyping of the 293 lotus accessions, and the genomic locations of these were obtained by mapping the primers to the lotus genome (GenBank assembly accession: GCA_003033685.1).

The genetic diversity parameters, including the allele number of each locus (Na), the effective number of alleles (Ne), Shannon’s information index (I), observed heterozygosity (Ho), expected heterozygosity (He), and Wright’s inbreeding coefficient (Fis), were calculated using POPGEN version 1.31. Another important parameter, polymorphism information content (PIC), was estimated using Cervus 3.0.

### 2.3. Population Structure and Kinship Matrix Analysis

With allelic data of the SSR markers, the population structure of the 293 lotus accessions was analyzed using STRUCTURE (version 2.3.4) with the admixture model. The hypothetical number of subpopulations (K) was set from 1 to 10. For each K, 7 independent runs were performed with a burn-in period of 10,000 iterations, followed by 100,000 iterations of Markov Chain Monte Carlo (MCMC) for each run; then, the population membership estimates (Q-matrix) of each K in each run were obtained. The obtained Q matrixes were processed with the STRUCTURE HARVESTER program, generating the maximum likelihood value (LnP(D)) and ad hoc quantity (ΔK). The best K value was then determined according to the distribution of LnP(D) and ΔK. Using the best K value, the Q matrix of the 7 independent runs was integrated by the CLUMPP (version 1.1), which was used for subsequent association analysis. Based on SSR marker data, the relative kinship coefficients (K-matrix) were calculated for each pair of lotus accessions using SPAGeDi (version 1.5a).

### 2.4. Association Analysis

Association tests between individual markers and phenotypic data of the five traits were performed using TASSEL (version 3.0), and the regional association analysis was conducted using the developed InDel markers within the genome regions of interest. The mixed linear model (MLM) was used to incorporate information of population structure (Q-matrix) and familial relationship (K-matrix) generated above and to estimate the association between markers and the five traits (*p*-value). To minimize the false-positive results, corrections for multiple testing were performed using the false discovery rate (FDR) and generated the adjusted *p*-value (Q-value). The marker–trait associations were considered as significant based on a threshold of *p* < 0.001 and Q < 0.05. The proportion of the phenotypic variance explained by a single associated marker (R^2^) indicated the fixed marker effects. In order to detect the reliability of the trait-associated markers, association analysis was conducted between each single marker and the phenotypic data of each trait in each year. To detect the effect of the key markers on PA, the significant differences of the PA-related traits corresponding to the different genotypes of each markers were analyzed using DunCan multiple-range test.

### 2.5. Identification of the Key Genes

In the corresponding candidate regions of the key markers, the genes (including the upstream 2 Kb) containing the SNPs and InDels, which showed distinct allelic distribution between the LA and SA lotus groups in our previous study and resulted in nonsynonymous mutations, were screened out. These genes were considered as the key genes underlying the major QTL and key markers associated with lotus PA.

## 3. Results

### 3.1. Phenotypic Variation

As the samples representing the varieties of lotus in China, the PA phenotypic data showed a wide range of variation, including PTH (30.40–167.2 cm), PDH (38.00–197.20 cm), LL (16.72–63.35 cm), LW (11.90–53.05 cm), and FD (9.90–31.80 cm) in the four-year measurement. The variation coefficient ranged from 20.50% to 25.25% for PTH, 20.02% to 23.37% for PDH, 20.04% to 31.94% for LL, 21.28% to 24.72% for LW, and 18.66% to 21.07% for FD, respectively (Table 1). The phenotypic data of the five traits all showed normal distributions (Figure S1), indicating that this panel was suitable for association analysis on the PA-related traits. No significant difference for each trait was found among the four-year phenotypic data revealed by one-way statistical analysis (Table 1). The heritability of PTH, PDH, LL, LW, and FD was 0.94, 0.92, 0.98, 0.99, and 0.93, respectively (Table 1). The high repeatability and heritability indicated that the phenotypic variance was mainly genetically controlled in the population, making it suitable for QTL mapping.

For the correlation analysis, all the PA-related traits showed significant positive correlations with each other (*p* < 0.001) (Table S2). In addition, correlation coefficients between LL and LW (0.980 to 0.982) were higher than other pairs in all the four years’ analysis, followed by the pair of PTH and PDH (0.913 to 0.944) (Table S2).

### 3.2. SSR Polymorphisms and Genetic Diversity

In total, 93 pairs of SSR primers generated discernible, polymorphic, and reproducible bands (Table S3). All of the SSRs were well distributed across the eight lotus chromosomes, and their coverage ranged from 7 SSRs on Chr3 to 18 SSRs on Chr1 (Table S3). Based on these SSRs, a total of 349 reliable alleles were identified, and all of them were polymorphic. The average number of polymorphic alleles per SSR was 3.753, ranging from 2 to 9 (Table S4). For the evaluation of the genetic diversity of the population, the mean value of the PIC was 0.441, ranging from 0.114 to 0.813 (Table S4). In addition, the I, which also reflected the polymorphism of the markers, ranged from 0.240 to 1.869, with a mean of 0.881 (Table S4). There results suggested that all the markers showed a moderate level of polymorphism. Ho refers to the ratio of the actual number of heterozygotes in a genomic locus to the total number of individuals in a population, He refers to the ratio of the expected number of heterozygotes in a genomic locus to the total number of individuals in a population, and Fis is used to quantify the deficiency (1 > Fis > 0) and excess of heterozygotes (0 > Fis > −1) in the genomic locus. In our study, the mean value of the Ho was 0.392, lower than that of the He value (0.515). The mean Fis was positive (0.232) (Table S4), which, as well as the analysis of Ho and He, indicated an excessive homozygosity in the natural population of lotus.

### 3.3. Population Structure and Relative Kinship

**Figure 1:**
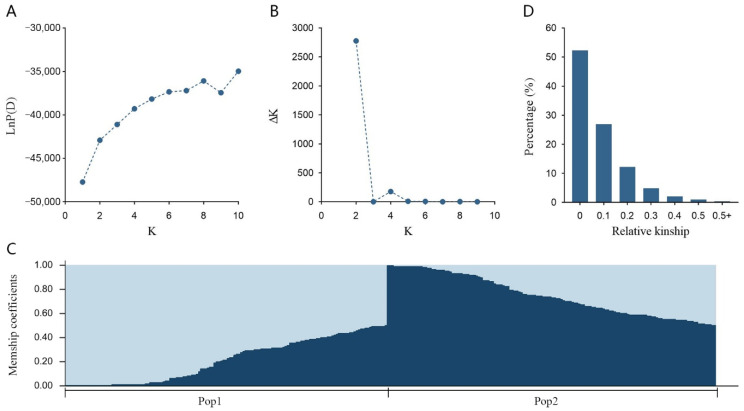
Population structure analysis and kinship coefficients distribution of 293 lotus accessions estimated by 93 genomic-SSR markers. (**A**) The most likely number of subpopulations (K) by LnP(D) analysis; (**B**) the most likely number of subpopulations (K) by ΔK analysis; (**C**) the 293 lotus accessions were classified into two subpopulations, Pop1 (light blue zone) and Pop2 (dark blue zone); (**D**) the distribution of pair-wise kinship coefficients between 293 lotus accessions.

The underlying genetic structure can generate false-positive results in the association mapping analysis, so estimation on genetic structure was necessary prior to the association mapping analysis to avoid false-positive results [32]. Using the selected 93 SSR markers, population structure (Q-matrix) and kinship coefficients (K-matrix) were estimated, which enhanced the statistic power in association analysis. STRUCTURE analysis showed that LnP(D) gradually increased along with K from 1 to 10, with no obvious cutoff point (Figure 1A), while the ΔK showed a strong peak at K = 2 (Figure 1B), indicating the presence of two subpopulations (Pop1 and Pop2) in the entire population (Figure 1C). To better discriminate the members from the subpopulations, the membership coefficient was set at 0.70 as a threshold to divide the members into three groups: group 1 (G1), group 2 (G2), and admixed group (AD) (Table S5). With a membership coefficient greater than 0.70 in subpopulation 1, 85 genotypes were assigned into G1, including 80 *N. nucifera* and 5 interspecific hybrids. With a membership coefficient greater than 0.70 in subpopulation 2, 83 genotypes were assigned into G2, including 7 *N. nucifera* and 26 interspecific hybrids. The rest of 125 genotypes with varying levels of membership shared between the two subpopulations were assigned into AD, including 100 *N. nucifera* and 25 interspecific hybrids (Table S5). The corresponding Q matrix (Table S5) was used for subsequent structure-based association analysis.

The construction of pairwise kinship matrix demonstrated that the kinship estimates ranged from 0 to 0.96, with a mean of 0.034 (Table S6). More than half (52.57%) of the pairwise kinship values were equal to 0, while 92.65% of the values were < 0.2 in the population (Figure 1D). These results indicated that the relatedness level between most accessions in the population was weak, which might be due to the broad range collection of genotypes.

### 3.4. InDel Maker Development in the Candidate Regions

In our previous research, a total of 386 InDels located in the candidate genomic regions were screened out [29]. Among these InDel loci, 160 InDels widespread in the candidate regions were selected to design primers for the polymorphism detection through PCR amplification. At last, 51 InDel markers which produced discernible, polymorphic, and reproducible bands were confidently scored (Table S7). Then, we genotyped the population using the 51 InDel markers.

In total, 117 reliable alleles were identified, and all of them were polymorphic. The mean number of the polymorphic alleles per primer pair was 2.294, ranging from 2 to 4 alleles (Table S8). The PIC values ranged from 0.209 to 0.490, with an average of 0.355, and the I values ranged from 0.441 to 0.976, with a mean value of 0.665 (Table S8). The mean value of Ho was 0.351, lower than that of the He 0.443, while the mean value of Fis was 0.210 (Table S8). These results suggested that there is an excess of homozygosity within the candidate genomic regions.

### 3.5. Marker Trait Associations

**Figure 2:**
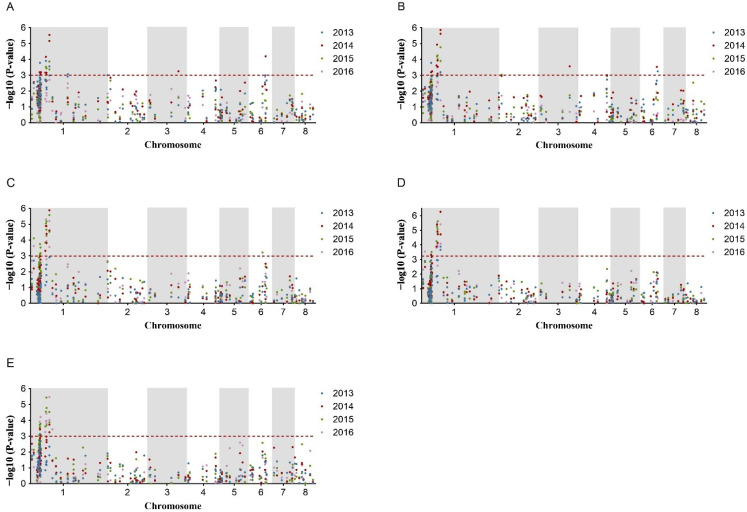
Manhattan plots of SSR and InDel markers associated with PA-related traits in the 8 chromosomes of lotus (*Nelumbo* Adans.). (**A**) Petiole height, (**B**) peduncle height, (**C**) leaf length, (**D**) leaf width, and (**E**) flower diameter. The blue, red, green, and purple dots represent the results of association analysis in 2013, 2014, 2015, and 2016, respectively. The horizontal red dashed line indicates the threshold with significant differences at −log10 (*p* value) = 3. Chromosomes and physical position of SSR and InDel markers were on the *X*-axis.

In order to screen the key loci associated with PA, association analysis was performed using the 144 molecular markers (93 SSRs and 51 InDels) and the phenotypic data of the five traits in four years. MLM model was used for association analysis by taking Q matrix and K matrix into account. At the threshold of *p* < 0.001(−log10 (*p*-value) >3.0) and Q < 0.05, a total of 125 significant associations were screened out from 2013 to 2016, among which 65 marker–trait pairs were unique (Table 2, Figure 2). For the 65 marker–trait pairs, 39 of them, accounting for 60.00%, were repeatedly observed in at least two years (Table 2, Figure 2).

These significant associations covered 26 molecular markers (8 SSRs and 18 InDels), and the average proportion of the phenotypic variance explained by an individual marker was 8.61%, ranging from 5.05% to 18.16% (Table 2). The amount of the markers associated with PTH, PDH, LL, LW, and FD was 14, 12, 12, 11, and 16, respectively (Table 2). Among these associated markers, 17 markers (4 SSRs and 13 InDels), accounting for 65.38%, were associated with at least two PA-related traits (Table 2). Notably, there were six markers (SSR067, NNIndel_81, NNIndel_94, NNIndel_95, NNIndel_99, and NNIndel_101) simultaneously associated with all of the five PA-related traits, forming a total of 30 marker–trait pairs of interest. Among the 30 marker–trait pairs, 28 of them showed interannual repetitiveness (Table 2). This was in agreement with the significant phenotypic correlation among the five PA traits and indicated the pleiotropic effect of these markers.

**Figure 3:**
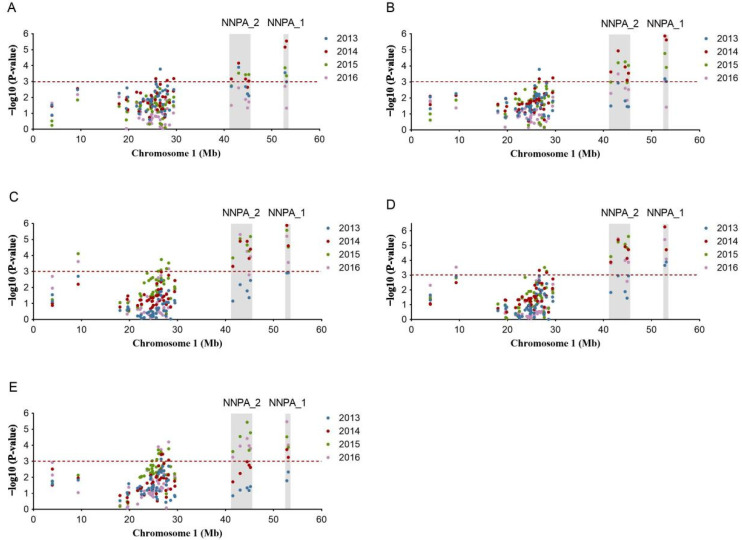
Manhattan plots of SSR and InDel markers associated with PA-related traits within the candidate regions on Chromosome 1 in lotus (*Nelumbo* Adans.). (**A**) Petiole height, (**B**) peduncle height, (**C**) leaf length, (**D**) leaf width, and (**E**) flower diameter. The blue, red, green, and purple dots represent the results of association analysis in 2013, 2014, 2015, and 2016, respectively. The horizontal red dashed line indicates the threshold with significant differences at −log10 (*p* value) = 3. The shadow lines represent the fine-mapped interval NNPA_1 and NNPA_2.

As InDel markers were derived from candidate regions, all of the 18 detected InDels were concentrated in 9–54Mb of Chr1, while the 8 detected SSRs were separately distributed on 3 chromosomes, and one of them was located in 9–54Mb Chr1, exactly the same as the 18 InDels location. Therefore, this region on Chr1 became the emphasis of our follow-up research. From the Manhattan plot of 9–54Mb Chr1, the association mapping showed that two intervals, named NNPA_1 and NNPA_2, displayed remarkably higher −log10 (*p*-value) for all the five PA-related traits at a strict threshold of −log10 (*p*-value) = 3.0 (Figure 3), which are the potential regions for the discovery of key genes controlling PA traits.

### 3.6. Identification of Key Markers and Genes

**Figure 4:**
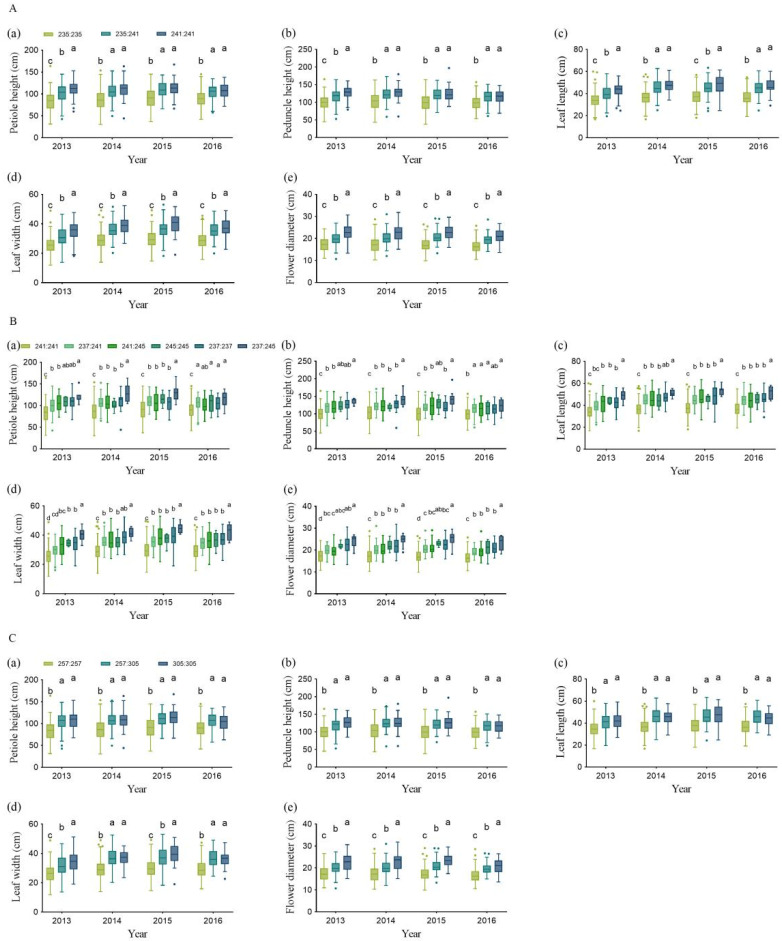
Phenotypical analysis of the PA-related traits regarding the different genotypes from 2013 to 2016. Three, six, and three genotypes were present, corresponding to NNIndel_99 (**A**), SSR67 (**B**), and NNIndel_81 (**C**), respectively. (**a**–**e**) represent the five PA-related traits. Different letters in lower case on the top of the bars indicate significant differences at *p* < 0.05 tested by DunCan multiple-range test using SPSS.

Within NNPA_1 region, there were two markers, NNIndel_99 and SSR067, and they were both associated with all of the five PA-related traits (Figure 3). Moreover, these two markers showed a much higher degree of association with the five traits than other markers in the four-year analysis. NNIndel_99 showed the strongest association with PDH, LL, and LW in 2013 and 2014, with FD in 2014 and 2016, and with PTH in 2015 (Table 2). It explained 6.70–8.76%, 6.02–10.04%, 9.89–10.42%, 6.92–11.79%, and 6.31–10.55% of the phenotypic variation for PTH, PDH, LL, LW, and FD, respectively (Table 2). There were three genotypes (235:235, 235:241, and 241:241) regarding the alleles of NNIndel_99 present in the association panel (Figure 4A), which indicated the genetic effects of NNIndel_99 on PA-related traits (Figure 4A(a–e)). Phenotypic analysis of these three genotypes in the population revealed that all the PA-related parameters corresponding to 235:235 were significantly lower than those of the other two genotypes (235:241 and 241:241). In contrast, the genotype of 241:241 showed the largest PA, significant differences of two PA-related traits (leaf width and flower diameter) between 235:241 and 241:241 were observed, and no significant difference for the other three PA-related traits was found between 235:241and 241:241 (Figure 4A(a–e)). The other key marker SSR067 explained 8.39–12.54%, 8.50–12.72%, 9.62–11.55%, 10.43–11.97%, and 8.03–10.76% of the phenotypic variation for PTH, PDH, LL, LW, and FD, respectively (Table 2). There were six genotypes (241:241, 237:237, 241:237, 245:241, 245:245, and 245:237) regarding the alleles present in the association panel (Figure 4B). Phenotypic analysis of these six genotypes in the population revealed that all the PA-related parameters corresponding to 241:241 were significantly lower than those of the other five genotypes (Figure 4B(a–e)). In contrast, the genotype of 237:245 showed the largest PA, and the PA of the other four genotypes (237:241, 241:245, 245:245, and 237:237) showed different levels of fluctuation from 2013 to 2016 (Figure 4B(a–e)). Taking together the association analysis, the NNPA_1 locus was considered as an effective locus in controlling PA in lotus.

Within NNPA_2 region, there were five markers (NNIndel_94, NNIndel_81, NNIndel_95, NNIndel_96, and NNIndel_101) significantly associated with the PA-related traits (Figure 3). Among these markers, NNIndel_81 showed the strongest association with PTH, PDH, LL, and LW, and it explained 6.42–7.37%, 5.50–8.39%, 8.31–10.10%, and 5.55–10.43% of the phenotypic variation for PTH, PDH, LL, and LW, respectively (Table 2). Therefore, NNIndel_81 was selected as the key marker for subsequent analysis. Three genotypes (257:257, 257:305, and 305:305) regarding NNIndel_81 were found in the association population (Figure 4C). Phenotypic analysis revealed that all the PA-related parameters corresponding to 257:257 were significantly lower than those of the other two genotypes (257:305 and 305:305). In contrast, the genotypes of 257:305 and 305:305 showed larger PA, and no significant difference was found between them (Figure 4C(a–e)). Finally, based on the results above, the NNPA_2 was also considered as an effective locus in controlling PA in lotus.

In our previous study, the candidate regions associated with PA traits were identified based on the genome resequencing results [29]. Therein, two of the candidate regions covered NNIndel_99 and SSR067 and contained 21 SNPs and 2 InDels, which showed distinct allelic distribution between SA and LA accessions (Table S9); one of the candidate regions covered NNIndel_81 and contained five SNPs, which also showed distinct allelic distribution between SA and LA accessions (Table S10). Subsequently, we focused on the genes, the amino acids encoded by which were altered due to the SNPs or InDels. Finally, one SNP was found to alter the amino acid sequence of gene LOC104590208 (3-oxoacyl-(acyl-carrier-protein) synthase, mitochondrial) (Table S9); another SNP altered the amino acid sequence of LOC104586303 (uncharacterized) (Table S10). Therefore, LOC104590208 and LOC104586303 were considered as the key genes for the regulation of PA traits.

## 4. Discussion

PA has a great influence on photosynthetic efficiency and the accumulation of plant biomass [33]. In addition, PA also affects the ways of application and the ornamental value of lotus [2]. However, the reports of genetic mapping for PA in lotus are limited. This resulted in a scarcity of molecular markers closely linked to the PA traits, as well as in a poor understanding of the genetic mechanism of these traits. Given this background, illustrating the genetic and molecular mechanisms underlying the major QTL and key markers associated with lotus PA is of great importance for lotus breeding.

### 4.1. Phenotypic Diversity and Heredity of PA-Related Traits in Lotus

For the purposes of association analysis, the ideal method is to establish a panel as genetically diverse as possible [34]. In this study, we evaluated the PA traits of 297 lotus accessions for four years and used these data to analyze the marker–trait association. Considerable phenotypic variations in the investigated traits were found in the population which were in accordance with normal distribution (Table 1, Figure S1). For each genotype, the phenotypic data of the PA traits were relatively stable from 2013 to 2016 (Table 1), and high heritability of PA-related traits detected in the association population indicated that the PA of lotus was a stable character under normal climate conditions. Thus, the PA traits were mainly controlled by genetic factors and could be regarded as a stable character in genetic or association analyses, which was suitable for association mapping.

### 4.2. Genetic Diversity

Detailed knowledge of genetic diversity in natural populations is important for understanding the forces responsible for evolutionary change [35]. Studies on genetic diversity in different lotus populations have been previously reported [36,37]. In a study on lotus genetic analysis, the mean value of He and PIC was 0.5 and 0.43, respectively, using 50 SSR makers and 92 genotypes [36], while Hu et al. [37] reported the mean value of He and PIC was 0.542 and 0.516, respectively, using 20 SSR makers and 50 genotypes. In our study, the genetic diversity of 293 lotus accessions were assessed based on 93 SSR markers, and the mean value of He and PIC was 0.515 and 0.441, respectively. Although these studies were conducted with different accessions and molecular markers, all the results revealed a moderate level of genetic diversity in the investigated populations. This may attribute to the long-term artificial selection for some important ornamental or agronomic traits during the breeding process, such as flower color, plant architecture, rhizome size, and so on. In addition, the asexual propagation depending on rhizomes and hybridization breeding based on limited elite accessions also restricted the genetic diversity. The excessive homozygosity observed in our study and Hu et al. [37] both verified the nonrandom mating within the lotus populations. Such homozygosity excess is also observed in previous analyses of *Camellia* [38], *Osmunda japonica* [39], and so on.

### 4.3. Genetic Structure and the Analysis Model

The genetic architecture in an association panel is prone to generating spurious associations; therefore, accurate evaluation of population structure is crucial for association mapping [40,41]. In a population structure analysis of 210 lotus accessions based on 38 SSRs, 16 sequence-related amplified polymorphisms (SRAPs), and 11 amplified fragment length polymorphism (AFLPs), three clearly distinct subpopulations were identified and, in general, corresponded to *N. nucifera*, *N. lutea*, and their hybrids [42]. In our study, although two theoretical subpopulations were detected statistically, both Pop1 and Pop2 contained *N. nucifera* and interspecific hybrids, and the major genetic origins of Pop1 and Pop2 (91.3% and 71.3%, respectively) were both derived from *N. nucifera*. In addition, high levels of phenotypic variability in the PA-related traits were found within each subpopulation (Figure 1). On the other hand, the mean kinship between all pairs was quite low (0.034). All the above indicated a weak population structure and made this lotus population applicable to association analysis in our study. Various mixed models could be used to perform association analyses, such as general liner model (GLM), GLM incorporating the Q-matrix (GLM (Q)), MLM incorporating the Q-matrix (MLM (Q)), MLM using the K-matrix as the sole random effect (MLM (K)), MLM using the Q matrix and the K matrix as the covariances (MLM (Q + K)), and so on. Compared with other models, MLM (Q + K) are more effective to avoid losing true associations and control false-positive associations in the association analysis, even though some traits were influenced by the population structure to some extent [43,44]. In our study, MLM (Q + K) ensured the accuracy of the association analysis results. This has been a powerful approach for improving the accuracy of associations in many cases.

### 4.4. Association Mapping Study

In lotus, only a few markers associated with PA have been identified by linkage mapping or association mapping. Yang et al. [19] reported the association mapping of four PA-related traits based on 129 lotus accessions and 98 SSR markers, and 11 markers were found to be associated with PA traits, which involved 25 SSR–trait pairs. In our study, 293 lotus accessions were used to carry out association mapping of the five PA-related traits using 93 reported SSR markers and 51 newly developed InDel markers. At the *p* < 0.001 and Q < 0.05 level, a total of 65 marker–trait pairs were identified, and the mean phenotypic variance explained by an individual marker was 8.61%, ranging from 5.05 to 18.16% (Table 2). Although 26 markers (8 SSRs + 18 InDels) were screened out with significant associations with phenotypic data, most of them could only explain the variance of the phenotypes by less than 10%, which suggested that PA-related traits were controlled by multiple genes with small effects in lotus. These results were in agreement with previous studies, which dissected the genetic architecture of PA in maize [45,46], cotton [16], and sorghum [17]. For the 15 SSR–trait pairs with significant associations detected in our study, only 4 of them were in common with the results in Yang et al. [19], and the other 11 were newly reported. The same original SSR markers were used for association analysis on the same PA traits between Yang et al. [19] and our study, but the association analysis results were not completely consistent. The difference in the results may be explained by the different sample size, different accessions, different association analysis models, and different thresholds used in the two studies. Similar results were also observed in previous mapping studies in other plants, which found that different mapping populations detected different QTL regions for the same traits [12,47]. Interestingly, the four common SSR–trait pairs between Yang et al. [19] and our study were all related to SSR067, which was found to be significantly associated with PTH, PDH, LL, and LW. The same results in different studies indicated the reliable associations between SSR067 and PA traits of lotus. In addition to SSR067, 24 other marker–trait pairs were also identified repeatedly in different years from 2013 to 2016 in our study (Table 2). Repeatability of marker–trait associations over multiple years or environmental conditions is crucial in association mapping to discriminate false-positive associations [48,49]. Hence, the marker–trait pairs identified by different studies and showing interannual repeatability are ideal tools for marker-assisted selection (MAS) breeding. In our study, 17 markers were simultaneously associated with more than one PA-related trait, which was accordant with the significant correlation among the five PA-related traits, and also indicated a pleiotropic or colocalized association, as reported in the studies of chickpea [50]. Further effort is still needed to validate the robustness of these markers, especially under different genetic backgrounds and environmental conditions.

In addition, our study selected three important markers (two InDels and one SSR) from the genotype–phenotype association analysis by the chromosomal location and *p*-value, and the data showed that only one genotype for each marker definitely corresponded to lotus with SA, indicating that LA is a dominant character compared to SA in lotus. Moreover, SA lotus tended to be homozygous and showed low genetic diversity in each corresponding marker-linked locus (Figure 4), probably because of a long-term artificial selection of lotus with SA, while the LA character has undergone numerous ancestral recombination events and lacks selection pressure, which exhibits high genetic diversity. Hence, the lotus with SA exhibiting the homozygous genotypes and low polymorphism in the key markers are considered as ideal materials for QTL and gene mining. These results provided useful information for fine mapping and subsequent cloning of candidate genes regulating PA-related traits in lotus.

Some genetic maps with high density and resolution have been constructed in lotus [22,28,51], but there have been no reports of QTLs for PA of lotus, let alone fine mapping. It is still a challenge to identify causative QTLs and genes for complex traits. The availability of genome sequence data provided an opportunity to dissect candidate regions to discover their causative QTLs and genes. In this study, we applied a regional association mapping strategy to fine map PA-related QTLs. With this strategy, we identified two QTLs, NNPA_1 and NNPA_2, on Chromosome 1, which contained the selected markers with high −log10 (*p*-value) values. In the NNPA_1, two markers, NNIndel_99 and SSR067, were both strongly correlated with the PA-related traits and showed the highest −log10 (*p*-value) and phenotypic explanation. SSR067 was considered as a significant marker associated with PA-related traits in previous studies and the present study as mentioned above, which increased the reliability of SSR067 as an effective and true locus. Within the corresponding candidate regions of the two markers, a functional gene LOC104590208 encoding 3-oxoacyl-(acyl-carrier-protein) synthase (KAS) emerged as a potential candidate gene to control PA-related traits. KAS is a type II fatty acid synthase (FAS) and is the prime catalyst for fatty acid biosynthesis in plants [52]. Fatty acids are the basic components of cell or organelle membrane lipids, which are critical for plant development, cell signaling, abiotic stress responses, and pathogen defenses [53]. The mutation of homologous genes KASI in Arabidopsis exhibited a semi-dwarf phenotype, and the rosette leaves were much smaller than the wild type, accompanied with reduced fertility and impaired chloroplast division [54]. In rice, the OskasI mutant also exhibits a significant decrease in plant height, along with the short root phenotype [55]. Therefore, KAS is considered as an important gene for the regulation of lotus PA. The NNPA_2 region contained five significant markers associated with PA-related traits. In the corresponding candidate regions of the peak marker, a functional gene (LOC104586303) without annotation emerged as an important candidate gene for PA regulation. Further functional verification of both candidate genes (KAS and LOC104586303) will help to better understand the underlying genetic mechanism of the PA-related traits. The two major QTLs would be the important targets for map-based cloning.

Our study combined SSR and InDel markers to identify the effective QTLs and potential key genes associated with PA traits in lotus, which improved the confidence of the analyzed results. However, the limiting number of SSR markers (93) used in this study makes it very suboptimal for the association analysis. To increase the confidence, a power comparison between SSR and SNP measurement is recommended to conduct in the global relative differentiation, as used in Cortés AJ et al. [56]. Our experiment system provided perfect conditions for the lotus growth, which reflected the true phenotype of the PA traits. However, in the natural environment, lotus may face different environmental stresses, i.e., desiccation, extreme temperature, and floods, where the lotus PA trait may become plastic as a genetic adaptive strategy to cope with environmental challenges. No adaptive trade-offs of lotus PA were observed in our study. However, in the studies under uncontrolled conditions, the environmental effect should be added in the analysis together with genetic effect. Therefore, it would be interesting to find out whether the genes identified in our study associated with lotus PA traits play pleotropic effects when the plants are exposed to a challenging environment. For example, the genes identified by genetic mapping showed more functions in addition to the trait-associated function in common bean [57,58].

In the future research, verification of the gene function identified in our study is essential, which will not only facilitate the understanding of the genetic mechanism of PA traits in lotus, but also provide another avenue for lotus breeding [59]. The combination of forward genetic approaches and reverse genetic techniques will be utilized in more and more plant breeding work for the favored plant traits under complicated environmental conditions [60,61,62]. The relevant work is ongoing in our lab.

## 5. Conclusions

In the present study, we identified 26 markers significantly associated with PA-related traits using an association mapping approach. The 39 marker–trait pairs showing interannual repeatability in current mapping or confirmed in previous studies could effectively serve for marker-assisted breeding programs in lotus. Based on the significant markers, two PA-related QTLs and two candidate genes were preliminarily determined. Our study is the first attempt to fine map lotus quantitative traits by association mapping method. These results not only provide useful information for subsequent cloning of candidate genes controlling PA and elucidating the genetic basis of PA-related traits in lotus in the future, but also contribute to the development of varieties with target PA.

## Figures and Tables

**Table 1:** Phenotypic measurement of the PA-related traits of the 293 lotus accessions.

Trait	Year	Min	Max	Mean	SD	CV (%)	*p*-Value	H^2^
Petiole height (cm)	2013	31.30	163.80	94.25	23.84	25.29	0.013 *	0.94
	2014	30.40	163.20	95.43	23.94	25.09		
	2015	37.14	167.20	99.50	23.21	23.33		
	2016	41.96	145.14	96.88	19.86	20.50		
Peduncle height (cm)	2013	45.10	165.60	109.73	23.57	21.48	0.029 *	0.92
	2014	43.25	179.70	111.39	26.03	23.37		
	2015	38.00	197.20	109.96	25.58	23.26		
	2016	53.17	157.29	106.63	21.35	20.02		
Leaf length (cm)	2013	16.76	60.00	37.63	8.26	21.94	<0.000 **	0.98
	2014	16.72	62.73	40.76	8.36	20.51		
	2015	18.07	63.35	41.39	8.61	20.79		
	2016	19.15	60.65	40.69	8.15	20.04		
Leaf width (cm)	2013	11.90	51.22	29.23	7.23	24.72	<0.000 **	0.99
	2014	13.86	52.50	32.51	7.15	21.99		
	2015	14.64	53.05	33.29	7.53	22.61		
	2016	15.80	49.00	32.26	6.87	21.28		
Flower diameter (cm)	2013	10.70	30.60	19.00	3.79	19.95	0.021 *	0.93
	2014	10.28	31.80	18.76	3.95	21.07		
	2015	9.90	29.60	19.10	3.86	20.21		
	2016	10.50	28.60	17.96	3.35	18.66		

Min, minimum; Max, maximum; SD, standard deviation; CV, coefficient of variation; H^2^, broad-sense heritability. *p*-value, significance of difference among traits data measured in 4 years derived from one-way ANOVA analysis and the LSD test using SPSS. * means *p* < 0.05, ** means *p* < 0.01.

**Table 2:** The SSR and InDel markers significantly associated with the PA-related traits.

Marker	Trait	Based on Phenotypic Data of 2013			Based on Phenotypic Data of 2014			Based on Phenotypic Data of 2015			Based on Phenotypic Data of 2016		
		*p*-Value	Q-Value	R^2^ (%)	*p*-Value	Q-Value	R^2^ (%)	*p*-Value	Q-Value	R^2^ (%)	*p*-Value	Q-Value	R^2^ (%)
SSR35	Flower diameter				2.00 × 10^4^	1.29 × 10^2^	14.62	6.48 × 10^4^	7.60 × 10^3^	14.69			
SSR41	Petiole height	4.83 × 10^4^	1.56 × 10^2^	13.85	1.65 × 10^4^	5.33 × 10^3^	13.38						
	Peduncle height	8.78 × 10^4^	3.78 × 10^2^	13.14	4.90 × 10^4^	7.91 × 10^3^	12.27						
SSR67	Petiole height				2.84 × 10^6^	3.66 × 10^4^	12.54	4.34 × 10^4^	1.12 × 10^2^	8.92			
	Peduncle height	9.34 × 10^4^	3.01 × 10^2^	8.50	2.37 × 10^6^	1.53 × 10^4^	12.72	1.23 × 10^4^	3.18 × 10^3^	10.13			
	Leaf length				2.47 × 10^5^	7.96 × 10^4^	10.73	2.97 × 10^5^	7.66 × 10^4^	11.55	2.73 × 10^4^	5.88 × 10^3^	9.62
	Leaf width	1.30 × 10^4^	1.68 × 10^2^	10.43	1.90 × 10^5^	4.89 × 10^4^	10.95	1.92 × 10^5^	4.13 × 10^4^	11.97	8.05 × 10^5^	3.46 × 10^3^	10.82
	Flower diameter				5.67 × 10^4^	1.46 × 10^2^	8.03	1.33 × 10^4^	3.44 × 10^3^	10.24	9.55 × 10^5^	4.11 × 10^3^	10.76
SSR70	Flower diameter										3.69 × 10^4^	5.29 × 10^3^	11.92
SSR88	Petiole height				1.71 × 10^4^	4.40 × 10^3^	16.59				2.25 × 10^4^	2.90 × 10^2^	18.16
	Peduncle height				3.98 × 10^4^	7.33 × 10^3^	15.64						
SSR122	Petiole height	8.57 × 10^4^	2.21 × 10^2^	7.71									
	Peduncle height							9.08 × 10^4^	1.95 × 10^2^	8.24			
SSR165	Petiole height				3.75 × 10^4^	8.06 × 10^3^	9.08						
SSR319	Leaf width										5.72 × 10^4^	9.23 × 10^3^	6.05
NNIndel_66	Petiole height				6.56 × 10^4^	1.06 × 10^2^	5.31						
	Flower diameter							7.63 × 10^4^	8.20 × 10^3^	5.77			
NNIndel_68	Leaf length							9.70 × 10^4^	1.04 × 10^2^	5.50			
	Flower diameter							1.93 × 10^4^	4.15 × 10^3^	6.91	1.23 × 10^4^	2.65 × 10^3^	7.42
NNIndel_69	Flower diameter							3.27 × 10^4^	4.22 × 10^3^	6.47			
NNIndel_71	Leaf length							8.06 × 10^4^	9.45 × 10^3^	5.65			
	Flower diameter							2.60 × 10^4^	3.72 × 10^3^	6.66	1.87 × 10^4^	3.01 × 10^3^	7.07
NNIndel_72	Flower diameter				3.80 × 10^4^	1.22 × 10^2^	5.77						
NNIndel_73	Petiole height	1.66 × 10^4^	1.07 × 10^2^	7.14									
	Peduncle height	1.64 × 10^4^	2.11 × 10^2^	7.16	6.38 × 10^4^	8.23 × 10^3^	5.34						
	Leaf length							1.80 × 10^4^	2.58 × 10^3^	6.89			
	Leaf width				4.71 × 10^4^	7.60 × 10^3^	5.58						
NNIndel_74	Flower diameter				3.59 × 10^4^	1.54 × 10^2^	5.81						
NNIndel_78	Leaf length							6.68 × 10^4^	8.61 × 10^3^	7.73			
	Leaf width							3.02 × 10^4^	4.87 × 10^3^	8.46			
NNIndel_80	Leaf length							7.73 × 10^5^	1.42 × 10^3^	7.58	2.40 × 10^4^	6.19 × 10^3^	6.77
	Leaf width										2.89 × 10^4^	5.33 × 10^3^	6.63
NNIndel_81	Petiole height	1.27 × 10^4^	1.64 × 10^2^	7.37	6.92 × 10^5^	2.98 × 10^3^	7.00	2.96 × 10^4^	1.91 × 10^2^	6.42			
	Peduncle height				1.14 × 10^5^	4.90 × 10^4^	8.39	6.32 × 10^5^	2.72 × 10^3^	7.70	3.14 × 10^4^	4.05 × 10^2^	6.55
	Leaf length				1.29 × 10^5^	8.32 × 10^4^	8.31	8.90 × 10^6^	3.83 × 10^4^	9.40	4.89 × 10^6^	6.31 × 10^4^	10.10
	Leaf width				4.06 × 10^6^	2.62 × 10^4^	9.21	5.66 × 10^6^	2.43 × 10^4^	9.78	3.37 × 10^6^	4.34 × 10^4^	10.43
	Flower diameter							2.84 × 10^5^	1.22 × 10^3^	8.52	1.12 × 10^4^	2.90 × 10^3^	7.50
NNIndel_88	Flower diameter							9.31 × 10^4^	9.24 × 10^3^	7.52			
NNIndel_94	Petiole height				6.92 × 10^4^	9.92 × 10^3^	5.27						
	Peduncle height				2.38 × 10^4^	6.14 × 10^3^	6.08						
	Leaf length				4.83 × 10^4^	8.90 × 10^3^	5.56	1.42 × 10^4^	2.29 × 10^3^	7.08	4.62 × 10^4^	8.51 × 10^3^	6.23
	Leaf width				1.31 × 10^4^	2.42 × 10^3^	6.54	5.56 × 10^5^	1.02 × 10^3^	7.86	1.56 × 10^4^	3.36 × 10^3^	7.14
	Flower diameter							2.46 × 10^4^	3.96 × 10^3^	6.71	5.52 × 10^4^	7.12 × 10^3^	6.15
NNIndel_95	Petiole height				6.95 × 10^4^	8.96 × 10^3^	5.27	3.70 × 10^4^	1.19 × 10^2^	6.24			
	Peduncle height				1.15 × 10^4^	3.69 × 10^3^	6.63	5.57 × 10^5^	3.59 × 10^3^	7.81			
	Leaf length				1.31 × 10^5^	5.61 × 10^4^	8.30	2.22 × 10^5^	7.15 × 10^4^	8.63	5.49 × 10^5^	2.36 × 10^3^	8.02
	Leaf width				1.17 × 10^5^	5.04 × 10^4^	8.39	8.01 × 10^6^	2.58 × 10^4^	9.49	9.99 × 10^5^	3.22 × 10^3^	7.52
	Flower diameter							3.65 × 10^6^	4.71 × 10^4^	10.28	3.73 × 10^5^	2.41 × 10^3^	8.45
NNIndel_96	Peduncle height				7.71 × 10^4^	9.05 × 10^3^	6.94						
	Leaf length				1.54 × 10^4^	3.31 × 10^3^	8.31	5.25 × 10^5^	1.13 × 10^3^	10.06			
	Leaf width				7.28 × 10^5^	1.56 × 10^3^	8.93	1.44 × 10^5^	3.72 × 10^4^	11.24			
	Flower diameter							2.05 × 10^4^	3.77 × 10^3^	8.92	1.06 × 10^4^	3.43 × 10^3^	9.70
NNIndel_97	Petiole height				8.50 × 10^4^	9.97 × 10^3^	9.72						
NNIndel_99	Petiole height	2.79 × 10^4^	1.20 × 10^2^	6.70	6.87 × 10^6^	4.43 × 10^4^	8.76	1.37 × 10^4^	1.77 × 10^2^	7.04			
	Peduncle height	6.32 × 10^4^	4.07 × 10^2^	6.02	1.35 × 10^6^	1.74 × 10^4^	10.04	1.66 × 10^5^	2.14 × 10^3^	8.82			
	Leaf length				1.32 × 10^6^	1.70 × 10^4^	10.08	2.66 × 10^6^	3.43 × 10^4^	10.42	6.20 × 10^6^	4.00 × 10^4^	9.89
	Leaf width	2.16 × 10^4^	1.39 × 10^2^	6.92	5.35 × 10^7^	6.90 × 10^5^	10.79	5.40 × 10^7^	6.97 × 10^5^	11.79	3.90 × 10^6^	2.52 × 10^4^	10.30
	Flower diameter				1.85 × 10^4^	2.39 × 10^2^	6.31	2.95 × 10^5^	9.52 × 10^4^	8.49	3.34 × 10^6^	4.31 × 10^4^	10.55
NNIndel_101	Petiole height				9.30 × 10^4^	1.00 × 10^2^	5.05	3.62 × 10^4^	1.55 × 10^2^	6.25			
	Peduncle height				2.87 × 10^4^	6.18 × 10^3^	5.94	9.26 × 10^5^	2.99 × 10^3^	7.39			
	Leaf length				4.03 × 10^5^	1.04 × 10^3^	7.44	6.56 × 10^6^	4.23 × 10^4^	9.65	1.19 × 10^4^	3.85 × 10^3^	7.36
	Leaf width				1.87 × 10^5^	6.02 × 10^4^	8.03	2.38 × 10^6^	1.53 × 10^4^	10.52	1.34 × 10^4^	3.45 × 10^3^	7.28
	Flower diameter							1.64 × 10^5^	1.06 × 10^3^	8.99	1.58 × 10^4^	2.91 × 10^3^	7.21
NNIndel_104	Petiole height				6.48 × 10^4^	1.19 × 10^2^	5.32						
	Peduncle height				5.56 × 10^4^	7.97 × 10^3^	5.45						

## Data Availability

Supporting data are included within the article and its Supplementary Materials. Other relevant materials are available from the corresponding author upon request.

## Supplementary Materials

The following supporting information can be downloaded at: https://www.mdpi.com/article/10.3390/plants12061221/s1.
