# Supplementary material for: Identification of QTLs and Putative Candidate Genes for Plant Architecture of Lotus Revealed by Regional Association Mapping
Source: Plants (Basel). 2023 Mar 8;12(6):1221. doi: 10.3390/plants12061221 (PMC10051333; doi:10.3390/plants12061221)
Supplement: Supplementary file 1 [file plants-12-01221-s001.zip › Figure S1.pdf]

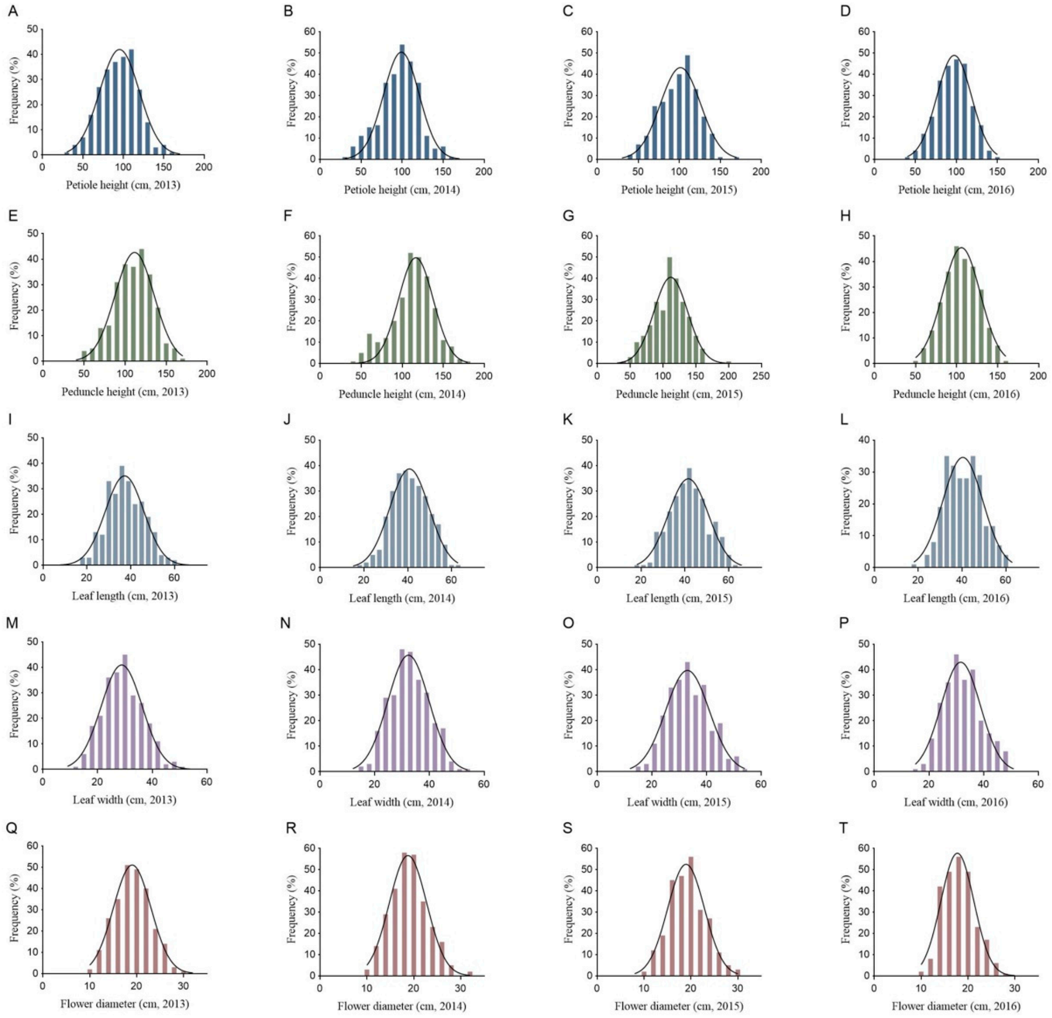

Figure S1 The distribution of accessions with the indicated level of petiole height (A-D), peduncle height (E-H), leaf length (I-L), leaf width (M-P), and flower diameter (Q-T). The values of each trait were evaluated in 293 lotus accessions in 2013, 2014, 2015 and 2016.
